# Membranous Croup—Diphtheritic Laryngitis

**Published:** 1901-01

**Authors:** S. A. Visanska

**Affiliations:** Atlanta, Ga.


### MEMBRANOUS CROUP—DIPHTHERITIC LARYNGITIS.*

By S. A. VISANSKA, M.D.,

ATLANTA, GA.

As this is the last meeting of the Atlanta Society of Medicine for the year 1900, I esteem it a great privilege to bring this paper before you for discussion, and while I claim little originality there might be some points new to a few and interesting to others. It is not my intention to take up all the etiology, symptoms, treatment, etc., but to bring out the principal points whereby we can distinguish this dreadful and death-dealing disease from the milder form, and then take up the treatment. Thanks to medical science and the improvement in the manufacture of surgical instruments, the death-rate has been brought down to a minimum. The bacteriologist tells us that in treating membranous croup we are dealing with the Klebs-Lœffler bacillus of diphtheria, so that we will not consider further the etiology, except to state that other organisms (the streptococci) are found along with the Klebs-Lœffler. Being called to a case the first thing we must do is to distinguish between false or spasmodic croup and true or membranous croup, and from the history of the case and symptoms presented you are to decide. Spasmodic croup usually comes on at night and suddenly, cough is dry and barking, there is hoarseness, child is restless, wants to be taken up and held upon the arm, little or no fever, and with proper treatment in from twelve to twenty-four hours the patient is entirely well. In membranous or true croup, of course the only positive proof would be the finding

---

* Read before the Atlanta Society of Medicine, December 20, 1900.

of the Klebs-Lœffler bacillus, but in laryngeal diphtheria it is difficult to obtain the membrane unless coughed up; constitutional symptoms are usually absent, partly on account of the protection afforded by the very numerous mucous glands, and partly on account of the absence of lymphatic glands and the supply of lymphatic vessels. These vessels connect with the bronchial glands. The main danger comes from the mechanical obstruction to respiration and the extension of the disease to the bronchi. In uncomplicated cases of membranous laryngitis excluding the ascending ones, there is little or no fever, the onset of the disease is gradual and it grows progressively worse; there is hoarseness, and after a short time complete aphonia; the stenosis is at first slight and only on inspiration; as the disease progresses the inspirations become more hurried, with dilatation of the ala nasi, recession of the chest, sometimes we even get the barrel-shaped chest; the skin and mucous membrane is of a blue or leaden hue, there is great restlessness, and the eyes seem to have a bulging appearance and the face a most pitiful expression, almost begging for oxygen. If nothing is done to relieve the obstruction, in a short time the child dies from asphyxia. Such is the picture usually presented to us at the bedside, and in what way are we to relieve these little sufferers?

*Treatment.*—When I am called to see a case of croup, and usually the call comes in the evening, unless I find the symptoms warrant a more radical cure, I treat them all alike; that is, for false croup I order the following prescription: calomel gr. ¼, salol gr. 1, soda bicarb. gr. ½, every half-hour until six doses are taken. For the croupy cough and relaxing effect I give apomorphia mur. gr. 1, and acid mur. dil. m. 30, syr. senega ʒ 6, syr. pruni. virg. ℥ 1½, aqua q. s. ℥ 4. M. Sig.: A teaspoonful every two hours for a child three years of age. Or if you prefer one of the ammonia preparations give mur. ammo. grs. 64, syr. ipecac ʒ 3, syr. pruni virg. ℥ 1½, aqua q. s. ℥ 4. M.: Sig. A teaspoonful every two hours. While some one goes for the medicine I place the child under a tent and pass medicated steam from a Holt's croup kettle for an hour or longer. The medication consists of carbolic acid, turpentine and oil of eucalyptus. The carbolic acid is placed in the water, the eucalyptus, or turpentine on the sponge as in the apparatus shown here.

One steaming usually suffices if you are dealing with false croup, but in membranous croup it has to be repeated according to circumstances. The croup kettle which I show you is very convenient, can be carried from place to place; it holds enough alcohol to burn six hours, and by using wood alcohol the cost is little. It possesses a great advantage over the older methods of conveying steam, as the apparatus is brought to the side of the bed and conducts the steam wherever you like. The old methods of conveying steam from a kettle placed on a stove or fireplace is rather inconvenient and inefficient.

When you return in the morning and find your patient very little or no better, the cough of a deep barking tone, hoarseness progressing, face cyanotic, then what is the next step? By all means use antitoxin and give a large dose, say for a child three years of age I do not hesitate to give 2,000 units, and then if it is necessary to repeat the dose you can give a smaller one. The reason I order a large dose at the beginning is the blood supply is smaller than to the tonsils and soft palate, therefore it takes longer to neutralize the poison and cause detachment of the membrane. In the meantime the child should receive concentrated nourishing food, and if the pulse give indications of a weak heart strychnine and whisky should be given; it is surprising the amount of whisky these little patients can stand. If there is constipation repeat the calomel and use enemas to cleanse the lower bowel. After using this treatment suppose we find in place of the membrane becoming absorbed, respiration easier, and hoarseness decreasing, it is just the reverse with symptoms of aphonia, hurried respiration, face cyanotic, retraction of the ribs, etc., what step shall be taken next? We must choose between two operations, tracheotomy and intubation. My mind is so made up between the two that I seldom consider the former, though bear it in mind in case circumstances call for it. Intubation or the procedure for the relief of dyspnea depending upon croup was first employed by Bouchut of Paris, in 1858. He used a hollow metallic cylinder about an inch in length, which was pressed into the larynx and allowed to remain, and had attached to it a silken thread to facilitate its removal and prevent it passing down into the trachea. The results of Bouchut's cases were not sufficiently satisfactory to recommend its general adoption and the procedure fell into disuse. In 1880, Dr. Joseph O'Dwyer, of New York, after numerous experiments upon dead subjects in the autopsy room of the New York Foundling Asylum, finally reintroduced this operation as a means of dealing with dyspnea resulting from laryngeal stenosis. Numerous modifications of the tube were made, and it is due to the careful work of O'Dwyer that the operation has become recognized as a legitimate procedure in the treatment of the symptoms arising from laryngeal obstruction. Having performed intubation successfully several times, can recommend it to every physician, and with proper fitting tubes believe that little injury can be done. With the proper amount of experience the operation is over in a few minutes, the child breathes easier, cyanosis disappears, and in a short while the child falls asleep almost exhausted. Before performing intubation it might be well to give a small dose of chloral and sodium bromide to reduce the amount of spasm sometimes caused by the introduction of the tube to prevent the tube being coughed up. The instruments I show you are the latest made by Ermold of New York. The tube can remain in the larynx for seven or ten days or longer without any discomfort to the patient, as the longer it remains the more accustomed the larynx is to the foreign body. After inserting the tube the question arises how to feed the child; if you give the food with the child sitting upright the food will run into the larynx, causing the child to cough and possible expulsion of the tube; to overcome this difficulty the head of the child should be held over the nurse's arm, and lower than the body, so that the food will run into the pharynx; only concentrated food should be given and even then it should be semi-solid. Ice cream is the ideal nourishment, but milk and soft rice, custard and gelatine can be given in this way. Sometimes you will find a few patients so sensitive that food cannot be given in this manner, then we must resort to rectal feeding. Before the introduction of antitoxin treatment by inhalation was almost universally adopted, and the fumes most destructive to the Klebs-Lœffler bacilli are those generated by the sublimation of calomel, and even to this day many physicians use it with good results. The child should be well wrapped up so that the face is free, thus exposing the least possible surface of the skin to the action of the mercury. It should then be placed in an ordinary croup tent and the calomel sublimed in such a manner as to fill it with fumes. The best apparatus is the steam spray or kettle which I show you; the boiler is replaced by a strip of tin or tin plate, upon which the calomel is placed. Another good arrangement, in case you do not have the kettle apparatus, is to put a small alcohol lamp in the bottom of an ordinary chamber and cover it with a pie pan or strip of metal to hold the powder. Usually forty to fifty grains of calomel are sufficient, and it should be burned every two or three hours, according to circumstances. It is not necessary to wake the child for treatment, and if the smoke causes much cough and irritation, lower the gas flame so that sublimation will take place less rapidly. It usually takes about ten minutes to sublime fifteen grains, and if care be taken that the calomel be pure there will be very little irritation. This treatment was first suggested by Corbin of Brooklyn. The attendant should be careful to inhale as little of the vapor as possible, as it is surprising how frequently the nurse becomes salivated and how seldom the patient is at all affected. To sum up the treatment of membranous croup we have antitoxin, intubation, steam inhalations, strychnine and whisky, calomel fumigations, nourishing food, and keep the gastro-intestinal canal clean.

